# *Rickettsia massiliae* and *Rickettsia conorii* Israeli Spotted Fever Strain Differentially Regulate Endothelial Cell Responses

**DOI:** 10.1371/journal.pone.0138830

**Published:** 2015-09-22

**Authors:** Jeremy Bechelli, Claire Smalley, Natacha Milhano, David H. Walker, Rong Fang

**Affiliations:** Department of Pathology, University of Texas Medical Branch, Galveston, Texas, United States of America; University of Minnesota, UNITED STATES

## Abstract

Rickettsiae primarily target microvascular endothelial cells. However, it remains elusive how endothelial cell responses to rickettsiae play a role in the pathogenesis of rickettsial diseases. In the present study, we employed two rickettsial species with high sequence homology but differing virulence to investigate the pathological endothelial cell responses. *Rickettsia massiliae* is a newly documented human pathogen that causes a mild spotted fever rickettsiosis. The “Israeli spotted fever” strain of *R*. *conorii* (ISF) causes severe disease with a mortality rate up to 30% in hospitalized patients. At 48 hours post infection (HPI), *R*. *conorii* (ISF) induced a significant elevation of IL-8 and IL-6 while *R*. *massiliae* induced a statistically significant elevated amount of MCP-1 at both transcriptional and protein synthesis levels. Strikingly, *R*. *conorii* (ISF), but not *R*. *massiliae*, caused a significant level of cell death or injury in HMEC-1 cells at 72 HPI, demonstrated by live-dead cell staining, annexin V staining and lactate dehydrogenase release. Monolayers of endothelial cells infected with *R*. *conorii* (ISF) showed a statistically significant decrease in electrical resistance across the monolayer compared to both *R*. *massiliae*-infected and uninfected cells at 72 HPI, suggesting increased endothelial permeability. Interestingly, pharmacological inhibitors of caspase-1 significantly reduced the release of lactate dehydrogenase by *R*. *conorii* (ISF)-infected HMEC-1 cells, which suggests the role of caspase-1 in mediating the death of endothelial cells. Taken together, our data illustrated that a distinct proinflammatory cytokine profile and endothelial dysfunction, as evidenced by endothelial cell death/injury and increased permeability, are associated with the severity of rickettsial diseases.

## Introduction

Rickettsiae are Gram-negative obligately intracellular bacteria with a predilection for infecting vascular endothelial cells [1]. Rickettsiae primarily target the vascular endothelium of small and medium sized vessels leading to vasculitis and ultimately edema in vital organs. The typical clinical manifestations of infections caused by spotted fever group rickettsiae include fever, rash, and frequently *tache noire*, and progress to encephalitis and pneumonitis in severe infections [2]. Pathophysiological effects of rickettsial infection on endothelial cells include an increase in vascular permeability, vascular inflammation, and pro-inflammatory cytokine production [3,4]. *Rickettsia*-infected endothelial cells have been reported to produce several pro-inflammatory cytokines and chemokines such as IL-6 [5], IL-8 and MCP-1 [6]. These responses are associated with the activation of endothelial cells [7] and enhanced endothelial cell permeability [8–10]. Furthermore, human and mouse endothelial cells exhibit anti-rickettsial activity under cytokine stimulation including TNF-α, IFN-γ and RANTES activation. Until now, it has never been completely understood how to distinguish the endothelial cell responses accounting for host immunity from those contributing to disease pathogenesis during rickettsial infection. Answering this question is crucial for better understanding the endothelial cell pathobiology in severe rickettsioses.

The bacterial agents *Rickettsia massiliae* and *R*. *conorii* are two genetically related rickettsial species with significantly different virulence. *R*. *massiliae* and *R*. *conorii* chromosomes exhibit >98% identity in coding sequence [11]. Interestingly, the clinical consequences of infections caused by *R*. *massiliae* dramatically differ from those by *R*. *conorii*. Previously believed to be nonpathogenic, *R*. *massiliae* has been recently documented to cause human infections that have presented as mild spotted fever rickettsioses in Argentina, France, and Italy [12]. *R*. *conorii* is the etiological agent of Mediterranean spotted fever (MSF), which is considered as one of the most severe and life threatening rickettsial infections. Among four strains of *R*. *conorii*, the Israeli spotted fever strain of *R*. *conorii* (ISF) is believed to be the most virulent with a case fatality rate up to 32.3% in hospitalized patients [13]. Therefore, *R*. *massiliae* and *R*. *conorii* (ISF) were employed in the present study to investigate the contributions of endothelial cell responses to the pathogenesis of rickettsial diseases.

Moreover, *R*. *massiliae* and *R*. *conorii* (ISF) occur in the same geographic regions [14]. Because serological cross-reactivity occurs across spotted fever group rickettsiae [15] and the primary means of diagnosis is through serum antibody assays, the accurate distinction between infections caused by these two species requires the identification of the actual infecting bacterium. This cross reactivity between the species and the overlap in geographic distributions highlight the need to better understand the pathological differences between these rickettsial species. A correct diagnosis is critical to predicting the pathological complications that would arise due to infection, and would allow physicians to anticipate complications and the correct response in the clinic.

Vascular endothelial cells perform a number of functions required to maintain homeostasis. In response to inflammatory stimuli, endothelial cells can be activated to gain new functions such as displaying surface adhesion molecules and chemokines that lead to recruitment and activation of circulating leucocytes [16]. However, inflammation can also cause endothelial cell injury, which disrupts these processes and results in endothelial dysfunction and death. Endothelial injury may lead to impairment of the endothelial cell barrier that retains fluid, plasma proteins and leukocytes within the intravascular space, leading to vascular leakiness [17]. In order to illustrate the contribution of endothelial cell responses to the pathogenesis of, and immunity to rickettsial diseases, we compared the responses of human dermal microvascular endothelial cells, HMEC-1, by a highly virulent rickettsial species, *R*. *conorii* (ISF), and a less virulent rickettsial species, *R*. *massiliae*. We hypothesized that *R*. *conorii* (ISF), the causative agent of a severe spotted fever rickettsiosis, would cause a pathological response (endothelial dysfunction) including increased inflammatory cytokines and cell death, while *R*. *massiliae*, a pathogen causing a mild MSF-like illness, would activate endothelial cells to secrete chemokines for interacting with immune leukocytes.

## Materials and Methods

### *Rickettsia* culture and preparation

*R*. *conorii* (Israeli spotted fever strain) was obtained from the American Type Culture Collection (ATCC). For cell culture propagation, rickettsiae were cultivated and maintained in Vero cell culture. *R*. *massiliae* was cultured as described previously [18]. After homogenization, rickettsiae were diluted in a 10% suspension of sucrose-phosphate-glutamate buffer (0.218 mM sucrose, 3.8 mM KH_2_PO_4_, 7.2 mM K_2_HPO_4_, 4.9 mM mono- sodium glutamic acid, pH 7.0) and stored at -80°C until used. The concentration of rickettsiae was determined by plaque assay and quantitative real-time PCR, described below. Plaque assay for testing the quantity of viable rickettsiae in stocks was performed as described previously [19].

### Cell culture and infection

HMEC-1 cells first described by Ades et al. [20] were cultured in MCDB 131 medium (Gibco, Grand Island, NY) supplemented with L-glutamine (10 mmol/L; Gibco), mouse epidermal growth factor (10 ng/mL; BD Bioscience, San Jose, CA), hydrocortisone (1 μg/mL; Sigma Aldrich, St. Louis, MO), and 10% heat-inactivated fetal bovine serum. Cells were grown in a humidified incubator with 5% CO_2_ at 37°C and used at approximately 95% confluence. Cells were inoculated at an MOI of 5 based on real-time PCR data. Intracellular growth occurred in a humidified incubator with 5% CO_2_ at 34°C until times indicated for each experiment.

### Quantification of rickettsial loads by quantitative real-time PCR

The number of rickettsiae in infected HMEC-1 cells was determined by quantitative real-time PCR as previously descried [21]. We first incubated endothelial cells with of the same number of *R*. *massiliae* and *R*. *conorii* (ISF) for 3 hours to allow the adhesion and internalization of viable infectious bacteria. At the selected time points as indicated, the infected samples and uninfected controls were washed with PBS and incubated with DNase to remove the dead or unattached bacteria. After all the samples were collected, genomic DNA was extracted following the manufacturer’s guidelines (Qiagen, Valencia, CA). The final concentration of DNA was determined by NanoDrop Spectrophotometer (Thermo Scientific, Waltham, MA). Samples were then analysed by quantitative real-time PCR targeting the *Rickettsia*-specific citrate synthase gene, using primers C5 (forward–GAG AGA AAA TTA TAT CCA AAT GTT GAT) and C6 (reverse–AGG GTC TTC GTG CAT TTC TT), as described previously [21]. Serial dilutions of plasmids containing a single copy of a fragment of *R*. *conorii* (Malish 7 strain) citrate synthase gene were prepared. Real-time PCR assays were performed in triplicate using an iCycler IQ from Bio-Rad (Hercules, CA), with each reaction including a non-template control (NTC). The results were expressed as rickettsial citrate synthase copies per ng of DNA.

### Quantitative detection of inflammatory cytokines in infected endothelial cells

HMEC-1 endothelial cells were infected with *R*. *massiliae* or *R*. *conorii* (ISF) as described or left uninfected as a negative control. Total RNA was prepared using Qiagen RNeasy Mini kit (Valencia, CA) following the manufacturer’s recommendations. Reverse transcription (RT) was performed using isolated and DNase-treated RNA with Bio-Rad iScript cDNA synthesis kit (Hercules, CA). The cDNA samples were then tested for the expression of mRNA of cytokines by real-time quantitative RT-PCR using SYBR Green PCR Master Mix on an iCycler IQ (Bio-Rad, Hercules, CA). Samples were run in triplicate, and GAPDH was used as the housekeeping gene control. The sequences for the sense and antisense primers used to quantify mRNA were: GAPDH 5’-GCACCGTCAAGGCTGAGAAC-3’, 5’TGGTGAAGACGCAGTGGA-3’:

IL-8: 5’-AGGGTCTTCGTGCATTTCTT-3’, 5’-ACACTGCGCCAACACAGAAATTA-3’; MCP-1: 5’-TTTGCTTGAAGTTTCACTGGCATC-3’, 5’-GCTCATAGCAGCCACCTTCATTC-3’

The ∆∆Ct method was used for quantification with results expressed as the mRNA relative ratio (2^-∆∆Ct^) [22]. In brief, the change in mRNA levels was determined using the following formula: fold-change 2^[∆Ct(infected) -∆Ct(control)]^, where ∆Ct (control) = threshold cycle (Ct) for target gene mock infection—Ct for β-actin mock infection; ∆Ct (infected) = Ct for target gene infection—Ct for β-actin infection.

### Cytokine and chemokine concentrations

Culture supernatants from *Rickettsia*-infected cells and uninfected control samples were filter-sterilized and stored as aliquots at -80°C. The concentrations of IL-6, IL-8, and MCP-1 protein present in the supernatant were measured by ELISA using Ready-Set-Go ELISA kits (eBioscience, San Diego, CA), following the manufacturer’s instructions. The limits of detection of the ELISA for cytokine concentrations were as follows: IL-6, 2 pg/mL; IL-8, 2 pg/mL; MCP-1, 7 pg/mL. Samples were assayed in duplicate and are presented as the average of ≥ 3 independent experiments. Absorbance values were obtained using a VersaMax ELISA microplate reader (Molecular Devices, Sunnyvale, CA), and the chemokine concentration was calculated from values obtained within the linear range of the standard curve.

### Annexin V staining and flow cytometry

HMEC-1 cells were washed with PBS before the addition of 0.025% (wt/vol) trypsin (Gibco, Grand Island, NY) to detach and lift cells from the culture surface. Fresh medium was added to the suspension to inactivate trypsin, and the cells were centrifuged at 330 × *g* for 10 min at 4°C. The pellet was washed with precooled PBS. Cells were first stained using a fixable LIVE/DEAD Near-IR Dead Cell Stain Kit (Life Technologies, Grand Island, NY) in accordance with the manufacturer’s protocol. Annexin staining solution was prepared using the manufactures provided 10× Annexin V binding buffer (10%), annexin V-PE (2%), and water (88%), and incubated with 1×10^6^ cells for 15 min at room temperature in the dark. Cells were washed in binding buffer, fixed with 1% paraformaldehyde containing binding buffer, and stored at 4°C until subjected to fluorescence-activated cell sorting analysis. Flow cytometry was performed on ⩾10,000 cells with a FACSCalibur laser cytometer (Becton-Dickinson, BD Biosciences, San Jose, CA), and data analysis was performed using FlowJo software.

### Measurement of lactate dehydrogenase (LDH) release

Culture supernatants from *Rickettsia*-infected cells and uninfected controls were filter-sterilized, and used in the assay immediately. LDH release was measured using the CytoTox96^®^ Assay kit according to the manufacturer’s specifications (Promega, Madison WI). Briefly, 50 μl of supernatant was added to 50 μl of reconstituted substrate mixture and incubated for 30 minutes in the dark. After the addition of stop solution, the absorbance was read at 490 nm using a VersaMax ELISA microplate reader (Molecular Devices, Sunnyvale, CA), and % cytotoxicity was calculated as experimental LDH release/maximum LDH release. Maximum LDH release was generated by incubating HMEC-1 cells with 0.05% Triton X-100 for 2 hours at 37°C and filter sterilizing to remove cell debris.

### Measurement of transendothelial resistance (TER) by electric cell-substrate impedance sensing (ECIS)

To determine vascular permeability of HMEC-1 cells, electric cell-substrate impedance sensing was employed using the ECIS Model 1600R (Applied Biophysics, Troy, NY, USA) and 8W10E gold-coated electrodes. Electrode arrays were washed with 10 mM cysteine and coated with 0.1% gelatin (Sigma Aldrich, St. Louis, MO) before plating approximately 1 × 10^5^ cells per well. Cells were allowed to attach before connection to the ECIS monitoring system. The resistance was observed until stabilization with average values being approximately 1,700 Ω. HMEC-1 monolayers were then infected with *Rickettsia* at an MOI of 5 with fresh medium added every 72 hours until completion of the experiment. Resistance was measured at approximately 4-minute intervals and was calculated by averaging the resistance of ten electrodes in each well. Uninfected controls were analyzed in duplicate, while infected monolayers were studied in triplicate.

### Caspase-1 and caspase-4 inhibition

In order to determine the mechanisms of LDH release, we employed the irreversible inhibitors specific for caspase-4 (Z-LEVD-FMK, 30 nM) (AG Scientific, San Diego CA) and caspase-1 (Z-YVAD-FMK, 30 nM) (Enzo Life Sciences, Farmingdale NY). The inhibitor was added to a complete HMEC-1 monolayer for 4 hours before infection with *R*. *conorii* (ISF) at an MOI of 5. Medium was changed every 24 hours with fresh medium containing caspase inhibitors.

### Statistical analysis

For comparison of mean values of different experimental groups, the one-way analysis of variance was determined using GraphPad Prism software version 5.01. Posthoc group pairwise comparisons were conducted using the Bonferroni procedure and overall α level of significance of 0.05.

## Results

### Plaque formation and growth kinetics of *R*. *massiliae* and *R*. *conorii* (ISF) in Vero and endothelial cells

**Fig 1:**
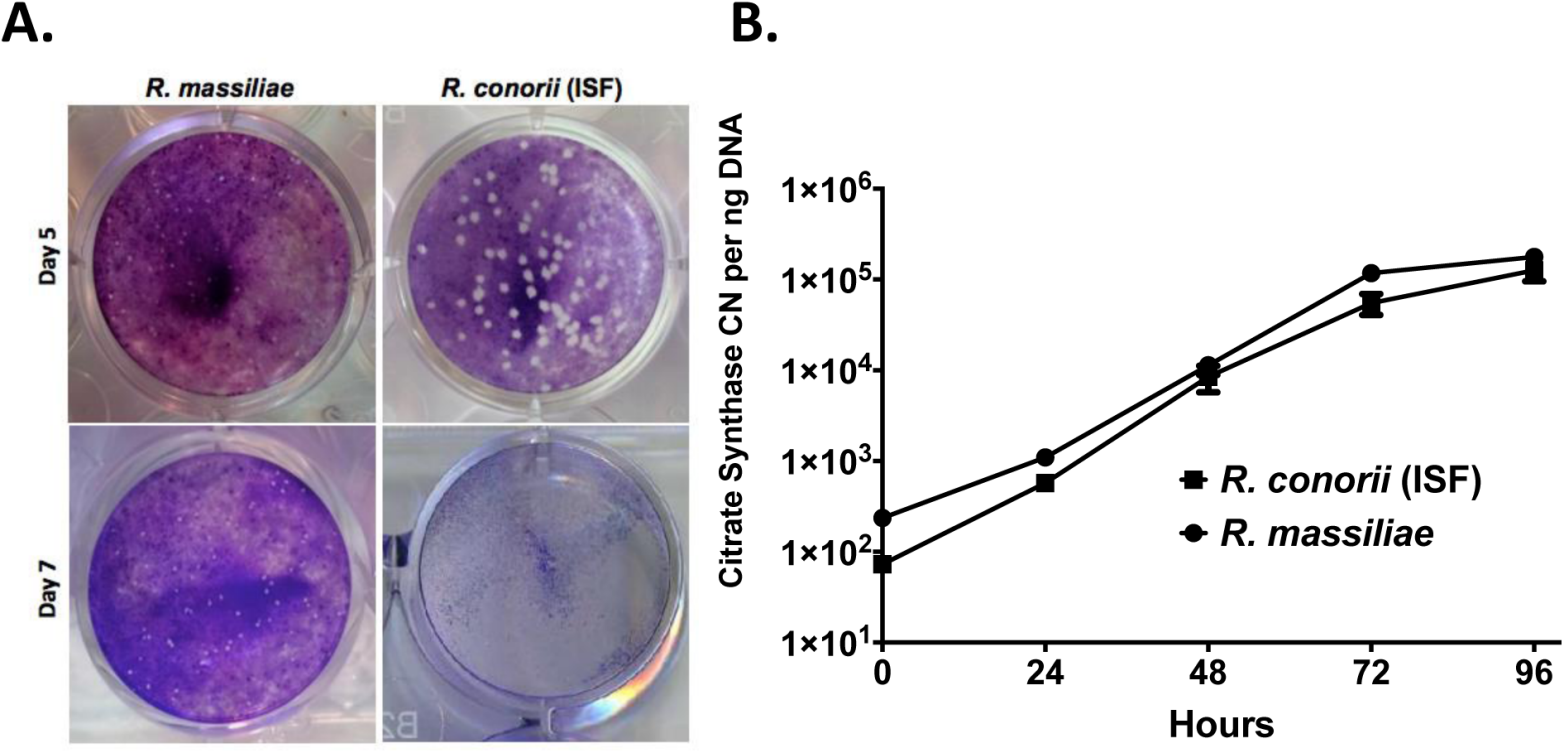
*R*. *massiliae* and *R*. *conorii* (ISF) infection resulted in plaques in Vero cell monolayers and replicated efficiently in the human dermal microvascular cell line, HMEC-1 cells. (A) *R*. *massiliae* and *R*. *conorii* (ISF) were grown in Vero cells at 34°C for 5 and 7 days to quantify the number of plaques present as revealed by crystal violet staining (B). *R*. *massiliae* and *R*. *conorii* (ISF) were grown in HMEC-1 cells for times indicated. Data are representative of three independent experiments (A and B).

To determine the entry and growth of *R*. *massiliae* and *R*. *conorii* (ISF) in eukaryotic cells, Vero cell monolayers were inoculated at an MOI of 5 and compared at days 5 and 7 post infection (PI) using standard overlay medium for the detection of plaques. Small foci of cell death were observed in monolayers infected with *R*. *conorii* (ISF) at day 3 PI (data not shown), increasing in size at day 5 PI, and by day 7 PI, the monolayer was completely destroyed (Fig 1A). Monolayers infected with *R*. *massiliae* showed no visible plaques at day 3 PI, and very small plaques became visible at 5 days PI. At 7 days PI, the monolayer infected with *R*. *massiliae* showed more plaques, and the size of each plaque was slightly larger than those at 5 days PI (Fig 1A).

To establish the growth kinetics of cultured *Rickettsia* species in human endothelial cells, confluent HMEC-1 cell monolayers were inoculated with an MOI of 5, and the total DNA was extracted at 24, 48, 72, and 96 hours PI. *Rickettsia massiliae* and *R*. *conorii* (ISF) were both able to invade and replicate in the HMEC-1 cells and grow with very similar kinetic curves up to 96 hours PI (Fig 1B). This finding allows for valid comparisons during infection by assuring that any differences observed in pathogenicity between these *Rickettsia* species would not be due to the quantity of bacteria.

### Differential regulation of cytokine and chemokine production in infected endothelial cells by *R*. *conorii* (ISF) and *R*. *massiliae*

**Fig 2:**
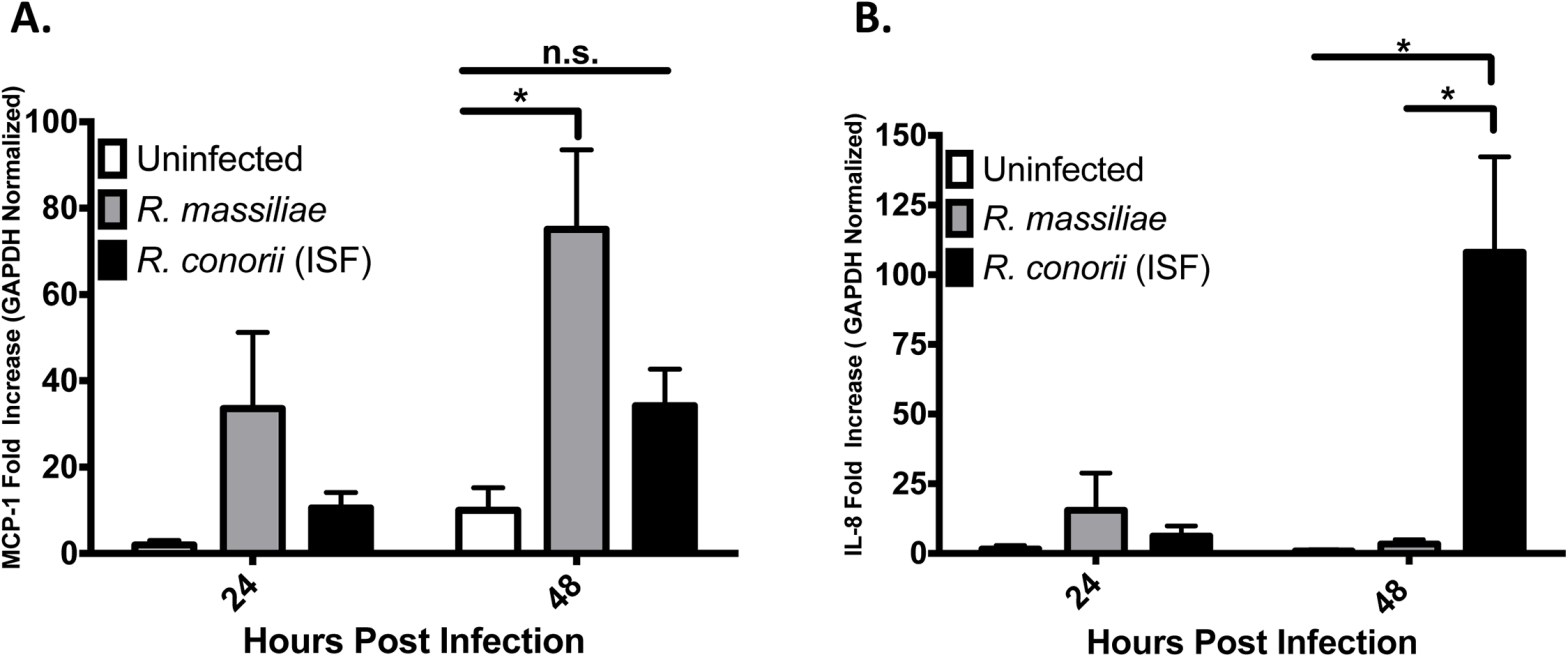
*R*. *massiliae* induced upregulation of MCP-1 mRNA levels and *R*. *conorii* (ISF) induced IL-8 mRNA levels in HMEC-1 cells. Real-time quantitative PCR analysis of the expression of MCP-1 mRNA (A) and IL-8mRNA (B) in HMEC-1 cells infected at an MOI of 5. Bar graphs indicate the average and standard error of three independent experiments. White bars represent uninfected cells, grey bars represent *R*. *massiliae-* infected cells, and *R*. *conorii* (ISF)-infected cells are represented by black bars. *, *p* <0.05.

To determine the potential differences in regulation of chemokine and cytokine production by endothelial cells infected with different species of *Rickettsia*, the levels of IL-8 and MCP-1 mRNA were quantified using RT-PCR at 24 and 48 hours PI. After 24 hours of infection, we did not observe any significant upregulation of MCP-1 or IL-8 mRNA in HMEC-1 cells infected by either species of rickettsiae compared to uninfected controls (Fig 2A and 2B). Interestingly, *R*. *massiliae*, but not *R*. *conorii* (ISF), induced a greater upregulation of MCP-1 mRNA at 48 hours PI compared to uninfected controls (Fig 2A). In contrast, IL-8 mRNA was dramatically upregulated in response to *R*. *conorii* (ISF), but not *R*. *massiliae*, at 48 hours PI (Fig 2B).

**Fig 3:**
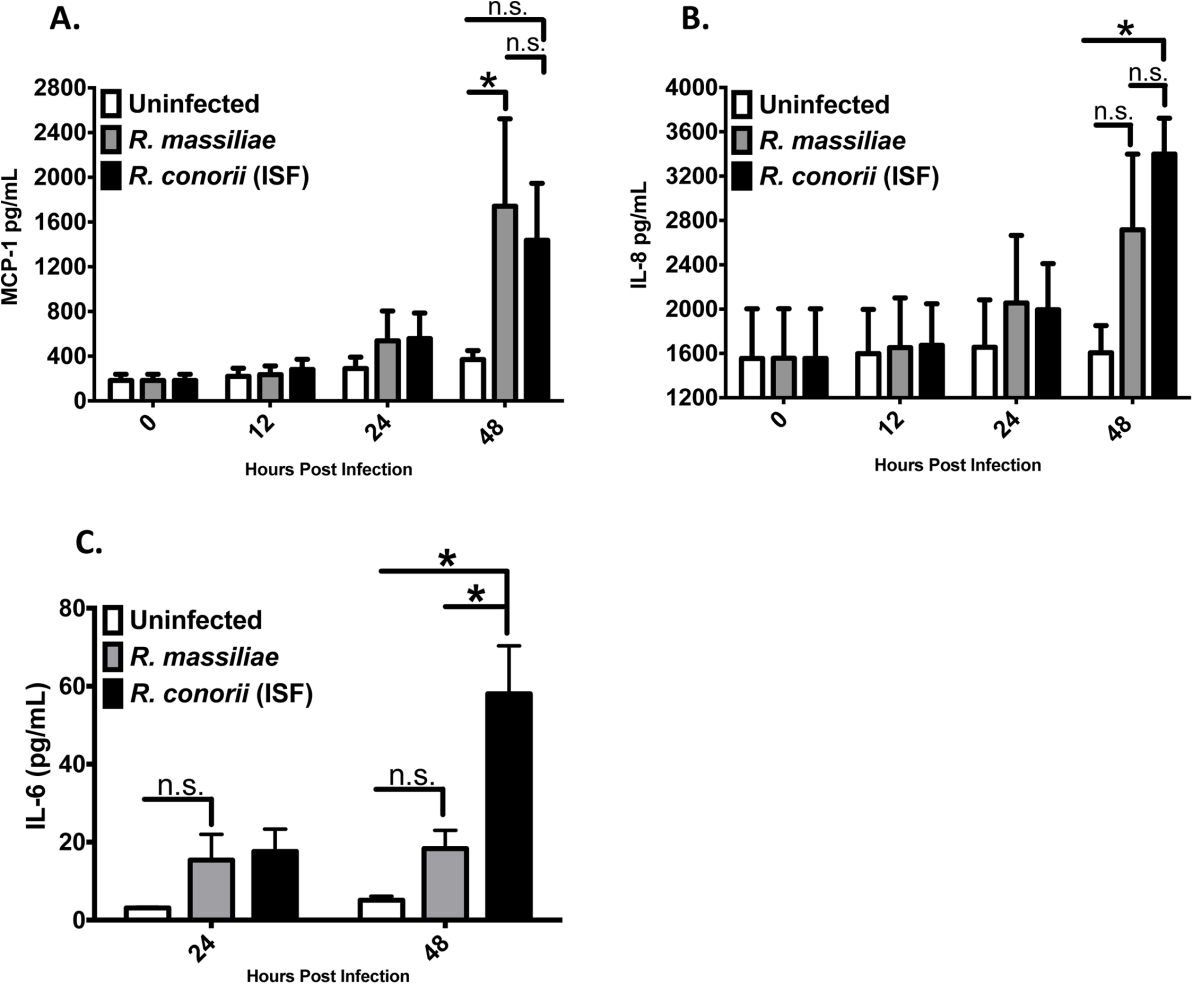
*R*. *conorii* (ISF) induced significant secretion of IL-8 and IL-6, while *R*. *massiliae* induced significant production of MCP-1 in infected HMEC-1 cells. The production levels of MCP-1 (A), IL-8 (B) and IL-6 (C) in the supernatant of *Rickettsia*-infected HMEC-1 cells was assessed by ELISA. Bar graphs indicate the average and standard error of three independent experiments. White bars represent uninfected cells, grey bars represent *R*. *massiliae*-infected cells, and *R*. *conorii* (ISF)—infected cells are represented by black bars. *, *p* < 0.05, ** n.s. = non-statistically significant.

To further evaluate the cytokine and chemokine secretion by endothelial cells infected with rickettsiae at the post transcriptional level, we performed ELISAs for MCP-1, IL-8, and IL-6. We did not detect any increased secretion of MCP-1, IL-8 or IL-6 by endothelial cells infected with either rickettsial species until 48 hours PI compared to uninfected controls (Fig 3A, 3B and 3C). After 48 hours PI, *R*. *massiliae* induced a higher level of MCP-1 in infected HMEC-1 cells compared to uninfected controls, while *R*. *conorii* (ISF) did not cause any significant secretion of MCP-1 in these cells (Fig 3A). Infection with *R*. *massiliae* did not induce increased secretion of IL-8 and IL-6 at 48 HPI compared to uninfected controls (Fig 3B and 3C). In contrast, *R*. *conorii* (ISF) induced significantly elevated levels of both IL-8 and IL-6 at 48 hours PI compared to uninfected controls. Furthermore, there is no significant difference in the amount of MCP-1 or IL-8 between these two rickettsial species (Fig 3A and 3B). The levels of IL-6 induced by *R*. *conorii* (ISF) were significantly greater than those by *R*. *massiliae* (Fig 3C).

### Endothelial cell death induced by *R*. *conorii* (ISF) at the late stage of infection

**Fig 4:**
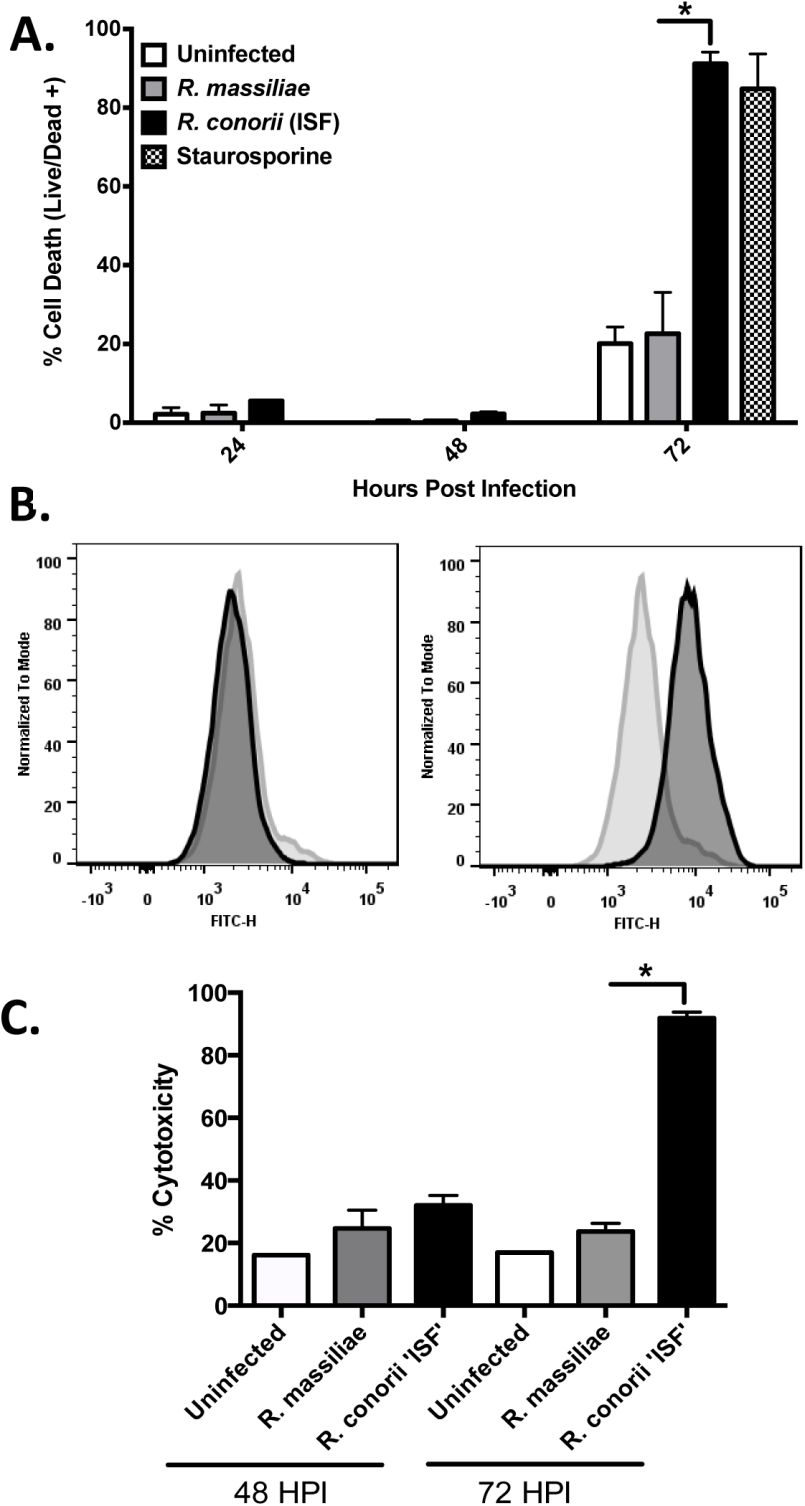
*R*. *conorii* (ISF), but not *R*. *massiliae*, induced cell death at 72 HPI in HMEC-1 cells: (A) Cells were infected at an MOI of 5 and stained with Live/Dead fixable dye for 30 minutes before fixation in paraformaldehyde. For flow cytometry experiments, ⩾10,000 cells were analyzed. (B) Annexin V staining of HMEC-1 cells following 72 hours of infection with *R*. *massiliae* or *R*. *conorii* (ISF). Cell counts were normalized to mode. (C) Lactate dehydrogenase (LDH) activity assay of monolayer supernatants demonstrating significantly increased levels of endothelial cell cytotoxicity after infection with *R*. *conorii* (ISF) for 72 hours. Bar graphs (A and C) indicate the average and standard error of three independent experiments. White bars represent uninfected cells, grey bars represent *R*. *massiliae*-infected cells, *R*. *conorii* (ISF)-infected cells are represented by black bars, and cells treated with staurosporine are represented by the checkered bar. *, *p* < 0.05.

Rickettsiae inhibit apoptosis of infected endothelial cells through NF-κB activation at 24 and 48 HPI [23, 24]. Here, we determined the viability of endothelial cells infected by each rickettsial species using Live/Dead® viability dye. In line with the previous studies, we did not find any significant increase in death of endothelial cells infected with either *R*. *massiliae* or *R*. *conorii* (ISF) compared to uninfected controls until 72 HPI (Fig 4A). However, at 72 hours of infection, cell death was identified in endothelial cells infected with *R*. *conorii* (ISF) (91%). Cells infected with *R*. *massiliae* showed levels of cell death similar to uninfected controls (Fig 4A). As a positive control, cells treated with staurosporine also showed significant cell death (85%). To confirm these results, we further determined the cell viability using annexin V staining. Interestingly, endothelial cells infected with *R*. *massiliae* for 72 hours showed no increase in the amount of surface exposed phosphatidylserine, which was detected by its binding to the protein annexin V. In contrast, *R*. *conorii* (ISF) induced a dramatic shift in the intensity of exposed phosphatidylserine compared to uninfected controls (Fig 4B).

To further investigate the endothelial cell death caused by *R*. *conorii* (ISF), we analyzed cell culture supernatants for the presence of LDH as an indicator of cytotoxicity. Following 48 hours of infection, no significant increase in cytotoxicity occurred in cells infected with either *R*. *conorii* (ISF) or *R*. *massiliae*. Interestingly, *R*. *conorii* (ISF) induced a significantly greater level of cytotoxicity at 72 HPI (92%) compared to uninfected controls (17%) (Fig 4C). In contrast, *R*. *massiliae* infection did not cause any significant LDH release from infected endothelial cells (24%).

### Increased endothelial cell permeability caused by infection with *R*. *conorii* (ISF) but not *R*. *massiliae*

**Fig 5:**
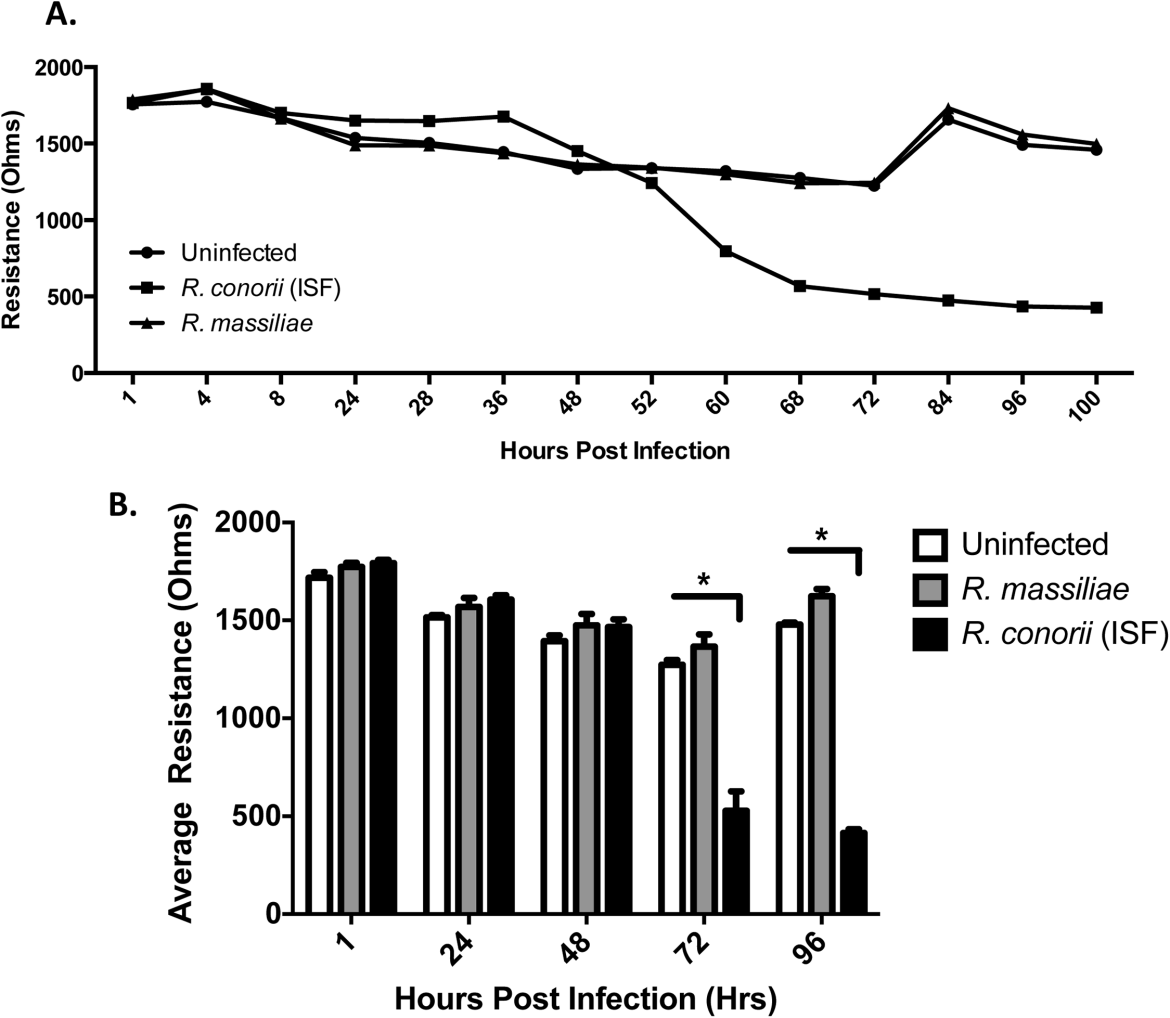
*R*. *conorii* (ISF), but not *R*. *massiliae*, increased endothelial cell monolayer permeability. (A) Representative ECIS graph demonstrating the loss of electrical resistance across an HMEC-1 monolayer in real-time. (B) Average resistance of endothelial cell monolayers after infection with rickettsiae for designated time points. Bar graphs (B) indicate the average and standard error of three independent experiments. White bars represent uninfected cells, grey bars represent *R*. *massiliae*-infected cells, and *R*. *conorii* (ISF)—infected cells are represented by black bars. *, *p* < 0.05.

To examine the effect of rickettsial infection on endothelial cell permeability, we used ECIS technology to measure TER in real time. Compared to uninfected controls, *R*. *conorii* (ISF) infection caused a marked decrease in electrical resistance across an HMEC-1 monolayer starting at 60 HPI up to 100 HPI (Fig 5A). In contrast, the cellular resistance in *R*. *massiliae-*infected endothelial cell monolayers did not show any significant difference from uninfected controls. Medium was changed at 72 HPI causing a spike in resistance before returning back to baseline levels near 1500 Ohms (Fig 5A).

To further determine the difference in endothelial cell permeability caused by different species of rickettsiae, we statistically compared the average resistance of these infected endothelial cell monolayers measured in three independent experiments. At time points consistently used throughout this study, there were no significant difference between *R*. *massiliae*-infected cells and uninfected controls (Fig 5B). At 72 and 96 hours of infection with *R*. *conorii* (ISF), there was a significant decrease in electrical resistance indicating increased endothelial monolayer permeability. Cells infected with *R*. *massiliae* demonstrated a trend of having increased resistance although this was not statistically significant. Thus, increased endothelial cell permeability caused by *R*. *conorii* (ISF) at 60 HPI is potentially associated with endothelial cell injury and/or death measured by dead-live cell staining, annexin V staining and LDH release at the late stage of infection.

### Endothelial cell death induced by *R*. *conorii* (ISF) is partially rescued by the inhibition of caspase-1

**Fig 6:**
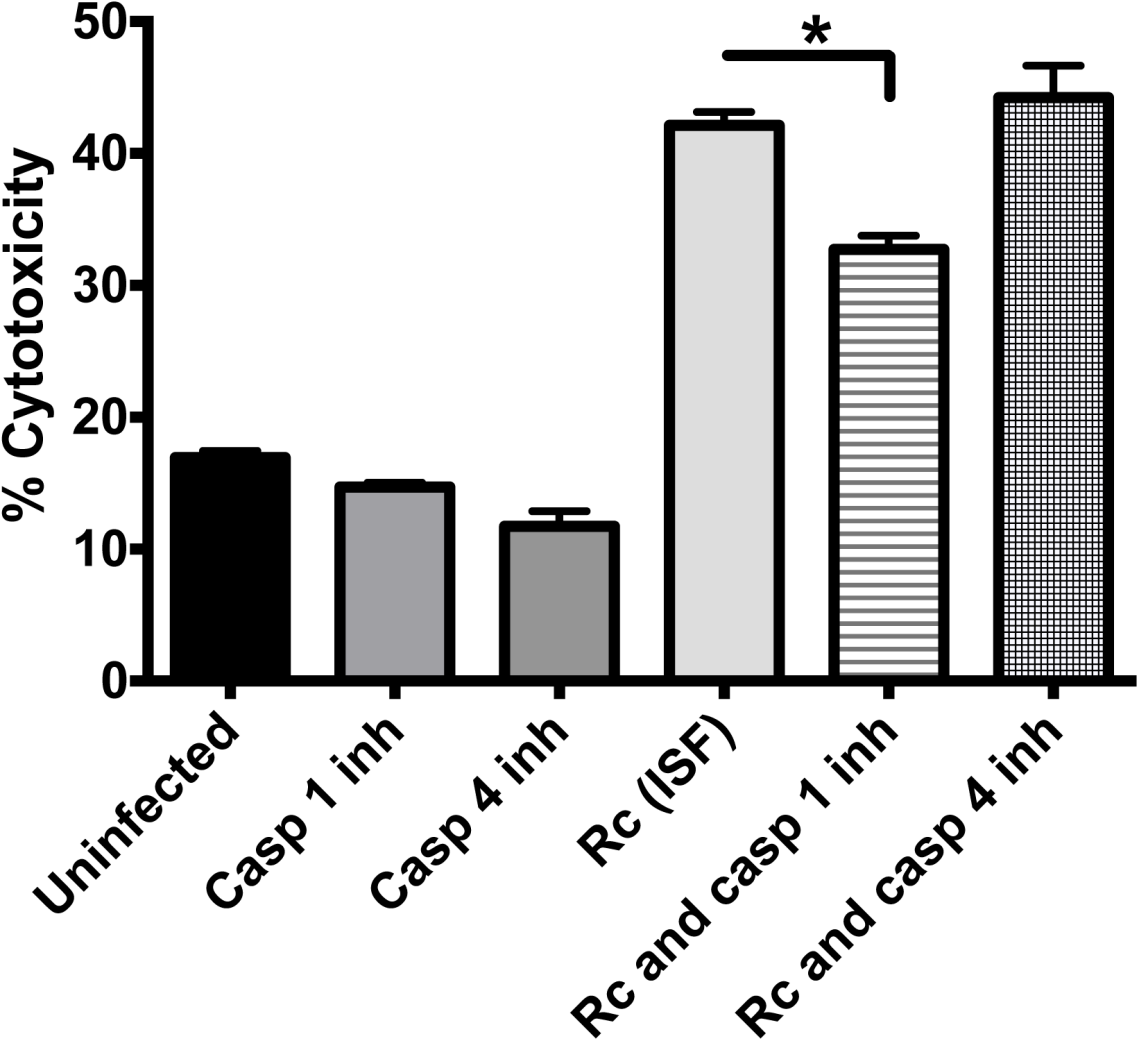
Endothelial cell death induced by *R*. *conorii* (ISF) is partially dependent on caspase-1. Cells were treated with caspase-1 and caspase-4 inhibitors and then infected with *R*. *conorii* (ISF) for 72 hours. Fresh medium and caspase inhibitors were added daily, and the removed supernatant was used immediately for the LDH activity assay. The bar graph indicates the average and standard error of three independent experiments. *, *p* < 0.05, inh = inhibitor, Rc = *R*. *conorii* (ISF), casp = caspase.

Since our results showed that *R*. *conorii*-induced endothelial cell death was characterized by a significant release of LDH activity, we further investigated the mechanisms involved in cell injury manifested as release of LDH by these infected HMEC-1 cells. It is known that caspase-1/4 mediates LDH release [25]. To examine the role of caspase-1 or caspase-4 involvement in cell injury in rickettsiae-infected endothelial cells, we examined LDH release caused by *R*. *conorii* (ISF) in infected HMEC-1 cells treated with inhibitors of caspase-1 or -4. We did not observe any significant change in LDH release in uninfected cells treated with inhibitors of caspase-1 or caspase-4 compared to untreated cells, which excludes the occurrence of non-specific effects of these inhibitors on cell injury and LDH release. Infection with *R*. *conorii* (ISF) caused a significant increase in LDH release compared to uninfected controls (42% and 17%, respectively), consistent with the results described in Fig 4B. Interestingly, as shown in Fig 6, the caspase-1 inhibitor significantly reduced the amount of endothelial cell cytotoxicity during infection with *R*. *conorii* (ISF) compared to cells without the inhibitor (33% and 42%, respectively). In contrast, caspase-4 inhibitor did not show a reduction in cytotoxicity (44%). These data suggest that caspase-1, but not caspase-4, partially mediates endothelial cell injury associated with LDH release.

## Discussion

*Rickettsia* are known to subvert host cell signaling to avoid cell death early in the infection by prolonging endothelial cell viability through inhibiting apoptosis in a NF-ĸB-dependent manner [26]. This impairment of programed death of the infected cells allows further intracellular rickettsial growth [27]. The later stages of infection involving increased endothelial permeability and cell death are poorly understood. To our knowledge, this study for the first time demonstrated that infection with highly virulent *R*. *conorii* (ISF), but not low virulence *R*. *massiliae*, led to endothelial cell injury and death, which was associated with increased endothelial permeability. Interestingly, we determined that endothelial cell injury/death caused by *R*. *conorii* (ISF) was mediated in part through caspase-1. Moreover, highly virulent *R*. *conorii* (ISF) induced a robust pro-inflammatory cytokine production profile by endothelial cells, including IL-8 and IL-6, while low virulence *R*. *massiliae* stimulated significant MCP-1 production by infected endothelial cells. These findings suggest that cell injury- and death-associated endothelial dysfunction and pro-inflammatory endothelial responses contribute to the pathogenesis of severe rickettsioses. Therefore, this new information sheds light on how pathological response of endothelial cells mediates the pathogenesis of severe rickettsioses.

Most strikingly, our studies illustrated that highly virulent *R*. *conorii* (ISF) induced endothelial injury and cell death during the late stage of infection measured by three independent approaches including annexin V staining, LDH release and fluorescent reactive amines dye (Fig 4A, 4B and 4C). In contrast, the endothelial cell injury and death were not observed in *R*. *massiliae*-infected cells throughout the course of infection *in vitro*. While annexin V staining is a marker of apoptosis, it also occurs in cells undergoing pyroptosis, an inflammatory process of caspase-1-dependent programmed cell death [28]. LDH is a soluble cytosolic enzyme that is released into the culture supernatant following the loss of membrane integrity that results from cell death [29]. LDH release is a useful marker of endothelial injury and was used recently alongside the activation of caspase-1 to distinguish pyroptosis from apoptosis [30, 31]. Most of the studies on endothelial cell survival promoted by rickettsiae focus on the early stage of infection [23, 26], although *R*. *rickettsii* was reported to cause cell death on days 5–6 PI [32]. Our research provided convincing evidence that rickettsiae caused endothelial cell death at the late stage of infection. This increased cell death may enhance rickettsial dissemination by forcing viable bacteria to invade neighboring or distant cells. Investigations relating to primary endothelial cell death *in vitro* and *in vivo* would provide enhanced understanding of rickettsial pathogenesis and endothelial cell dysfunction during infection.

What are the potential mechanisms by which rickettsiae cause endothelial cell death and injury? It has been considered that the pathogenesis of rickettsial diseases is dependent on bacterial growth, reactive oxygen species produced by endothelial cells, or rickettsial actin-based polymerization causing cell lysis [33]. Our results did not show any significant difference in endothelial cell infection rate and bacterial growth kinetics between highly virulent *R*. *conorii* (ISF) and low virulence *R*. *massiliae* (unpublished data and Fig 1B). Thus, our findings exclude the possibility that the difference in endothelial cell death and injury caused by these two species of rickettsiae is due merely to the intracellular growth of bacteria. Our study found that inhibition of caspase-1, but not caspase-4, significantly rescued the quantity of endothelial cell death (Fig 6). These results suggest that caspase-1 is, at least partially, involved in mediating endothelial cell death during rickettsial infection. However, whether the endothelial cell death caused by *R*. *conorii* (ISF) is pyroptosis or not requires further investigation. It is important to note that inhibition of caspase-1 rescued only a relatively small portion of cells, suggesting that mechanisms other than these associated with caspase-1 are involved in cell injury. It is important to mention that the levels of LDH released by *R*. *conorii* (ISF)-infected cells (Fig 4) was much higher than those shown in Fig 6. The culture medium in the experiments using caspase-1/4 inhibitors (Fig 6) was changed every day in order to maintain the concentration and the activity of the caspase inhibitors, while culture medium changes were not performed in experiments in Fig 4 allowing for a greater accumulation of LDH.

Fatal human rickettsioses are characterized by vasogenic cerebral edema and non-cardiogenic pulmonary edema resulting from increased endothelial permeability [34]. It is still not completely understood how rickettsiae cause increased microvascular permeability. Interestingly, only *R*. *conorii* (ISF)-infected human endothelial cell monolayers showed significantly increased permeability starting at 72 HPI, when death of these infected endothelial cells occurred (Figs 4 and 5). Endothelial cell barrier disruption or increased permeability has been reported to result from loss of endothelial cell adhesion molecules associated with cytotoxicity [35]. Recent studies also demonstrated that endothelial cell death is associated with pulmonary microvascular albumin leak in sepsis [36]. Thus, endothelial cell death likely plays a role in increased vascular permeability during infection with high virulent rickettsiae.

The secretion of cytokines and chemokines by *Rickettsia*-infected endothelial cells has been well characterized in *in vitro* infection models [6], although the effects of these inflammatory responses have never been fully understood. IL-6 and IL-8 have been proposed to be involved in the development of vasculitis induced by rickettsial infection and are secreted in large quantities from HUVECs infected with *R*. *conorii* (Malish 7) [37]. Oristrell et al. [5] reported that plasma IL-6 levels are significantly higher in patients diagnosed with MSF than in healthy controls (49.6 pg/ml vs. < 4 pg/ml). Additionally, plasma IL-6 levels are associated with more severe MSF (97.1 pg/ml vs. 32.7 pg/ml). These data are in agreement with Masueto et al, as IL-6 is a potent inducer of the acute phase response [5]. Damas et al. [38] observed that *R*. *conorii* induced a greater inflammatory response than *R*. *africae* as characterized by increased serum concentrations of IL-8, MCP-1, and soluble forms of adhesion molecules in patients with MSF than African tick bite fever. Clifton et al. [39] also showed that IL-8 is an important neutrophil and monocyte chemokine in *R*. *rickettsii* infection in human endothelial cells. Our present study suggests that severe rickettsioses are associated with endothelial pro-inflammatory cytokine production (IL-6 and IL-8), while less pathogenic rickettsial infection of endothelial cells correlates with increased levels of MCP-1. The cytokine profile in infections caused by these two closely related rickettsial species may have further implications in the differential diagnosis of these two rickettsial infections in the future.

Our research suggests that the severe form of Israeli spotted fever compared to the milder Mediterranean spotted fever-like illness caused by *R*. *massiliae* is likely due to the enhanced activation of endothelial cells leading to excessive inflammation and greater cell death, leading to increased vascular permeability and ultimately edema in critical organs and death. Because mutant rickettsial strains are unavailable, our study has relied on the comparison of two closely related species with distinct pathological consequences. This results in a limitation of the study in that the underlying mechanism responsible for bacterial pathogenicity are not completely understood. Moreover, several questions still remain unanswered regarding the exact mechanisms of cell death induced by rickettsiae such as the role of reactive oxygen species or lytic enzymes in cell injury. Other potential mechanisms of injury may be associated with the nature of the differences in bacterial pathogenicity. Ongoing work in our laboratory investigating the role of caspase-1 during rickettsial infections is shedding light on the pathogenic mechanisms of severe spotted fever rickettsial infections.
